# Where the public health principles meet the individual: a framework for the ethics of compulsory outpatient treatment in psychiatry

**DOI:** 10.1186/s12910-022-00814-8

**Published:** 2022-07-25

**Authors:** Sérgio M. Martinho, Bárbara Santa-Rosa, Margarida Silvestre

**Affiliations:** 1 Bioethics Institute, Faculty of Medicine, University of Coimbra, Azinhaga de Santa Comba, Celas, 3000-548 Coimbra, Portugal; 2 National Institute of Legal Medicine and Forensic Sciences, North Branch, Porto, Portugal; 3 Reproductive Medicine Department, Coimbra Hospital and University Centre, Coimbra, Portugal

**Keywords:** Involuntary treatment, Mental disorders, Ethical issues, Outpatient care, Psychiatry, Principle-based ethics

## Abstract

**Background:**

Compulsory treatments represent a legal means of imposing treatment on an individual, usually with a mental illness, who refuses therapeutic intervention and poses a risk of self-harm or harm to others. Compulsory outpatient treatment (COT) in psychiatry, also known as community treatment order, is a modality of involuntary treatment that broadens the therapeutic imposition beyond hospitalization and into the community. Despite its existence in over 75 jurisdictions worldwide, COT is currently one of the most controversial topics in psychiatry, and it presents significant ethical challenges. Nonetheless, the ethical debate regarding compulsory treatment almost always stops at a preclinical level, with the different ethical positions arguing for or against its use, and there is little guidance to support for the individual clinicians to act ethically when making the decision to implement COT.

**Main body:**

The current body of evidence is not clear about the efficacy of COT. Therefore, despite its application in several countries, evidence favouring the use of COT is controversial and mixed at best. In these unclear circumstances, ethical guidance becomes paramount. This paper provides an ethical analysis of use of COT, considering the principlist framework established by Ross Upshur in 2002 to justify public health interventions during the 2002–2004 severe acute respiratory syndrome outbreak. This paper thoroughly examines the pertinence of using the principles of harm, proportionality, reciprocity, and transparency when considering the initiation of COT.

**Conclusion:**

Ross Upshur’s principlist model provides a useful reflection tool for justifying the application of COT. This framework may help to inform sounder ethical decisions in clinical psychiatric practice.

## Background

### Introduction

Since the 1950s, Western psychiatry has moved towards deinstitutionalizing individuals with mental illness [1]. Indeed, the fall of the asylum movement progressively shifted the focus of psychiatric practice to community-based care settings. In several countries, the adoption of this community approach was accompanied by an extension of the legal frameworks to sanction the use of coercion to facilitate psychiatric investigations and treatment [2]. For example, Australia, Belgium, England, Portugal, and the United States are among the nations whose legislation contains the possibility of coercing an individual into receiving care [3].

Compulsory outpatient treatment (COT), also referred to as community treatment order, mandatory outpatient treatment, compulsory community treatment, or assisted outpatient treatment, is a modality of involuntary care that makes adherence to a treatment plan a legal obligation for an individual with mental illness in a community setting [4]. COT exists in over 75 jurisdictions worldwide, and its purpose lies in the governance of the legally mandated provision of care to those with mental illness and high levels of need [5, 6]. Specifically, COT authorises psychiatrists to stipulate treatment conditions with which the patient must comply under the penalty of returning to the hospital. The use of COT allows the doctor to formulate the patient’s treatment plan by deciding to which conditions the patient must adhere. Typically, these conditions involve taking the prescribed medication and attending appointments, and they may also extend to other areas of life if deemed necessary to achieve the therapeutic goals [5].

The arguments favouring COT focus on its perceived usefulness in clinical practice [7] and on patient protection, such in terms as of facilitating treatment compliance [8], promoting a less restrictive environment, reducing hospitalization, and preventing “revolving door” admissions [9]. Other positive justifications for COT include society’s obligation to care for individuals with mental illness. These arguments imply that patients with mental illness may lack judgement, insight, or decision-making capacities, which renders them inept at making autonomous decisions regarding their health [10]. Therefore, state intervention would be justified by its duty to protect patients with mental illness who are incapable of acting in their best interests.

The initiation of COT varies between countries. In nations such as England, Sweden, and Portugal, COT may only be implemented following hospitalization. In other jurisdictions, such as Scotland, the use of COT may take place directly in the community [3]. Despite these variations, the conditions for COT generally require the presence of a mental anomaly, a risk of harm to self or others, and a refusal to comply with treatment [11].

COT presents medical, legal, and social challenges, as well as ethical ones [10]. Nonetheless, ethical debates regarding compulsory treatment in psychiatry often centre on the principlist dichotomy of autonomy versus beneficence. This discussion tends to focus on the ethical justifications for and against the existence of compulsory treatment altogether, thus positioning the ethical debate at a preclinical level. However, the fact is that COT remains, for now, a legal instrument in several countries, and its use has increased in recent years [3, 12]. Furthermore, the current discussion regarding how to apply the Convention on the Rights of Persons with Disabilities (CRPD) [13] has increased the focus on COT because of several opposing interpretations, even within United Nations bodies [14]. These interpretations of how to apply the CRPD range from the need to abolish COT to the need for a capacity-based tests to be conducted for COT initiation [10]. As such, it does not seem plausible that these involuntary instruments will be prohibited in the near future [14].

Recently, public attention has been directed to the ethical justifications for and responsibilities of state interference in civil liberties to solve health crises. Due to the coronavirus disease 2019 (COVID-19) pandemic, large proportions of the world’s population, regardless of their health status, were forced to quarantine [15]. In 2002, Ross Upshur established a principlist framework to justify public health interventions based on an adaptation of Beauchamp and Childress’s standard principles [15, 16]. Upshur later exemplified the practice of these principles, applying them to ethically regulate the use of quarantine interventions during the 2002–2004 severe acute respiratory syndrome outbreak [17]. We suggest that this public health ethics framework may be adequate for guiding COT application.

As other authors have indicated previously [10], COT represents a severe restriction of a patient’s freedom and self-determination. However, its current existence and use cannot be ignored, especially because its evidence base is controversial at best. Amid the uncertainty regarding its role, COT warrants continued debate and analysis with the aim of shaping its implementation ethically. In this paper, we argue that Upshur’s principlist stance provides a useful framework to help individual clinicians navigate towards an ethical application of COT [16].

## Main body

### A necessary debate

Whether in inpatient or outpatient settings, compulsory treatment represents a significant interference with an individual’s basic rights and liberties. The use of COT means that an individual may be coerced to comply with actions that they would not otherwise engage in. The example of Portugal, where the Constitution had to be amended to integrate the Mental Health Law, illustrates the profoundness of this topic’s legal and ethical implications [18].

Indeed, COT is one of the most controversial topics in psychiatry [19]. Supporters of COT argue that this form of therapeutic enforcement promotes treatment compliance, potentiates the prevention of relapse, and reduces hospital admissions [6, 8]. Moreover, COT may constitute a less coercive means of action than hospitalization, particularly in those cases of “revolving-door patients” who have difficulty sustaining recovery and adhering to treatment plans and, thus, are frequently hospitalized. Furthermore, researchers speculate that COT may reduce the risk of harm to patients and others, as well as addressing the public’s fear of injury caused by such patients [3]. Additionally, ethical positions favouring COT are based on the notion that COT permits more sustained treatment, which is more desirable than frequent short-term hospitalizations [20]. Following on from this argument, others [2] highlight that both autonomy and beneficence are prima facie principles, and, consequently, the value of autonomy should not supplant the value of beneficence. Accordingly, protecting the patient’s health should have the same value as protecting the patient’s autonomy, and one should not be preferred over the other [2].

In psychiatric emergency situations, such as those requiring hospitalization, which may involve severe illness or imminent danger, it is apparent that beneficent measures should take priority over respecting autonomy [10]. Therefore, these conditions would justify the need to reduce autonomy. However, the need for autonomy restrictions in community-based settings is less apparent and is subject to several ethical reservations. For example, opponents of COT are concerned about the infringement of patients’ civil liberties and damage to the therapeutic alliance [19]. Another area of concern is the potential progressive change of COT from a liberty-enhancing instrument to a controlling treatment regime [11]. However, the most striking argument against COT arises from the lack of evidence regarding its efficacy, not only in terms of reducing readmission but also in improving quality of life [4, 21]. Despite some of the positive findings from Australian work groups about COT’s clinical outcomes [22], Burns and colleagues [23] concluded, in the largest randomised controlled trial (RCT) on the subject, the Community treatment orders for patients with psychosis (OCTET) trial, that community treatment orders did not reduce the rate of readmission of psychotic patients. Another analysis from the same study concluded that COT was not a cost-effective measure, as well as that COT duration was not associated with patients’ social outcomes even over the long term [21, 24].

As a result of this evidence, criticism has emerged regarding the limitations of RCTs for adequately evaluating the effectiveness of COT due to their conceptual, methodological, and analytical challenges [25]. For example, Mustafa [19] argued that RCTs may not be the gold standard for examining the effectiveness of COT due to their inability to recruit representative patients, thus suggesting that the OCTET study may not have captured the intended clinical population. Therefore, Mustafa [19] found that there are grounds to question the generalisability of the OCTET study’s negative results and that abandoning COT altogether may be a mistake.

Considering the current evidence, the effectiveness of COT appears controversial and mixed at the very best [6, 22]. Furthermore, there is disagreement regarding the most robust methodology to evaluate such intervention, resulting in a polarized debate between those who believe in the value of RCTs and wish to abolish COT and those who believe RCTs are a flawed method and call for the continuation of COT [10].

This lack of consensus regarding COT promotes ethical unrest. The foremost hesitancy regarding COT relates to the fact that, from a principlist view, the moral objective justifying the infringement of one principle over another must have a realistic prospect of achievement [26]. Therefore, in using COT, infringing autonomy in favour of a possibly ineffective practice raises legitimate questions.

Nonetheless, until further evidence-based clarification about the effectiveness of COT is presented, there is a need to ethically regulate this practice, which not only exists in several countries but is growing in use [27].

### A principle-led practice

Most people never experience a severe restriction of their autonomy regarding their health. Therefore, debates regarding the state’s ethical responsibilities in terms of imposing restrictions on individual freedoms for health purposes usually seem remote. However, considering the recent COVID-19 pandemic, the topic has resurfaced as an emergent issue, as several governments adopted policies restricting civil liberties in order to contain the virus’s spread. Opinions both justifying and condemning the restrictions emerged, and the debate continues about what constitutes admissible state action and interference in the individual sphere [28, 29]. Despite their differences in scale, some public health interventions—such as quarantine—and COT share the same dilemma, which is to understand the circumstances during which such liberty-restricting interventions are ethically justified.

To address the circumstances under which these interventions may be justified, Ross Upshur [16, 17] developed a modified principlist approach to support ethical intervention in public health, aiming to help implement quarantine ethically. He asserted that public health action must simultaneously meet four principles to be justifiable: (a) harm, (b) proportionality, (c) reciprocity, and (d) transparency. Despite recognizing the limitations of the principlist model, Upshur [16] defended its heuristic nature and its applicability to practice, stating it is not intended to be definitive.

Several authors have voiced reservations about the utility of principlism within psychiatry [26, 30]. Moreover, considering the lack of scientific consensus regarding COT, some authors argue that no general ethical justification for COT can be provided [5]. However, the same authors suggest that a justification based on the promotion of patient autonomy could represent a motive to utilize COT in some circumstances [5]. Principlism has previously been associated with COT; for exemple, Guillén [31] analysed COT through the perspective of the four classical bioethical principles, concluding that, as a therapeutic method, COT is consistent with the principles of respect for autonomy, justice, beneficence, and non-maleficence. However, Guillén’s analysis did not focus on guiding the practical use of COT. As such, we argue that Upshur’s set of principles is better suited to establishing a useful framework for the ethically justified application of COT. Additionally, this reflection on Upshur’s principles may give rise to concrete action to handle predicaments around compulsory interventions.

We consider each of the four principles identified above as they relate to the COT dilemma. In addition to this ethical reflection, we propose certain solutions regarding the application of these principles to COT.

#### The harm principle

The harm principle, enunciated by John Stuart Mill, establishes

That the only purpose for which power can be rightfully exercised over any member of a civilised community, against his will, is to prevent harm to others. His own good, either physical or moral, is not a sufficient warrant. ([32], p. 13)

In practice, the harm principle aims to ethically regulate clinical actions that will lead to harm avoidance, whether to self or others. Upshur [16, 17] stated that the harm principle constitutes perhaps the most elementary tenet for the implementation of quarantine. Indeed, in the situation of an imposed quarantine, the legitimacy and justification of the government’s actions would be based on preventing potentially infected people from spreading the disease.

Regarding the implementation of COT, legislation generally demands that the risk of harm to self or others is present. Despite this, if we consider Mill’s definition stated above, the harm principle would justify the use of COT only if there was a risk of harming others, not necessarily of self-harm. However, Stuart Mill delimitated certain exceptions to this justification; specifically, Mill contended that interfering with another’s actions is only legitimate when preventing harm to others, “unless [the person] is a child, or delirious, or in some state of excitement or absorption incompatible with the full use of the reflecting faculty” ([32], p. 13). This perspective suggests a possible justification for interfering on behalf of an individual’s own interests if they are a child or are in an altered state of mind that would impair their judgement. In this way, the harm principle seems to apply to not only preventing harm to others but, in certain circumstances, preventing harm to self. Therefore, Mill’s stance appears to match Beauchamp and Childress’s position, which stipulates that an absence of rationality or capacity to decide may justify medical intervention in line with the principle of beneficence—a position deemed as soft or justified paternalism [33].

Although the harm principle offers an ethical basis for applying COT to individuals with impaired judgement, it has limitations. An argument may emerge that, when there is significant risk of harm to self or others, a community setting may be inappropriate for implementing treatment, and, instead, hospital-based care may be necessary. Therefore, COT may prove an inadequate solution when the risk of harm is immediate. In COT circumstances, the COT justification of preventing patient harm or harm to others relates to a deferred rather than immediate harm prevention. However, this perspective may be problematic because the risk changes from a probable to solely a possible result, thus weakening the harm justification for the use of coercion.

The argument for the use of COT based on the harm justification is further weakened by the fact that the clinical predictive capacity for patient self-violence or violence against others is inadequate [34, 35], meaning that an unacceptable number of patients would have to be on COT to prevent harmful situations. The absence of harm immediacy undermines the use of the harm principle as justification for COT. However, despite playing a significant role, immediacy is not the sole component of the risk of harm. Therefore, the harm principle could still be used as justification for COT implementation, although it will demand from clinicians a much more thorough explanation compared to compulsory hospitalization. Furthermore, in this regard, excessive reliance on the harm principle to justify COT may contribute to stigma and validate the criminalization of those with mental illness through associating them with public danger and the concepts and language of the judicial system [8, 20].

In practice, given the dimensional—not categorical—aspect of the psychopathological approach, another question may emerge regarding how much risk for harm is sufficient to act to prevent harmful situations and whether there is a role for risk quantification in ethically legitimate coercion. In this regard, clinicians may be inclined to rely on risk assessment scales to support and justify a decision to implement COT. Although quantitative instruments may provide a useful method to systematically gather patient information and assess the risk of harm, risk quantification alone cannot ethically justify a clinical decision to initiate coercion.

Indeed, one fundamental aspect of an ethical decision is that it should produce an intended and predictable effect based on current evidence. However, quantitative instruments to assess the risk of harm cannot accurately predict or reduce self-harm or harm to others, especially in non-immediate situations and, thus, may not be better than clinicians’ predictions [36–38]. This inefficiency in the prediction of harm may be due to the low base rates of serious violence due to mental illness—these low base rates contrast with phenomena such as transmissible infections that may spread fast, which increases their incidence rates of potential harm [35]. Therefore, a decision to initiate coercion based on a risk assessment score that, ultimately, has no significant predictive value would be unethical and unjustifiable. Furthermore, with current instruments, such practice might add to the discrimination of individuals with mental illnesses, unjustified restrictions after false-positive predictions, and difficulties in access to care by those assessed as low risk [35]. As such, ethical evaluations of harm risk and the associated clinical decisions should focus on engagement with the individual patient, their specific issues, and circumstances rather than risk assessment scores [34].

In summary, the harm principle seems to contribute to the justification of COT but not without significant limitations. Clinicians should consider the argument’s hindrances and use it judiciously. The concept of harm, to self or others, must be concrete—not abstract or vague—and must have some quality of proximity. The potential for harm should not be used to justify the application of the harm principle. Additionally, the argument for COT based on the harm principle should be clear regarding what relevant value is being protected by restricting the patient’s autonomy and the link between the psychopathological phenomenon and a probable harmful result.

#### The proportionality principle

In its original formulation, Upshur [16] explained the proportionality principle as the principle of least restrictive or coercive means. In COT, this principle is best represented by the concept of proportionality itself because it encompasses a wider range of dimensions that need consideration. This principle comprises the prescriptive notion that higher degrees of liberty restriction should be accompanied by the need to protect significant human values, such as life or health. Therefore, restriction should not be used to serve values of lesser worth. The principle also recognizes that a wide range of means exists to achieve a proposed end and that these means should be applied progressively. Specifically, when considering these assumptions, coercion should be reserved only for cases where less restrictive methods have failed; hence, education, facilitation, and discussion should precede restriction or detention [16].

To initiate COT, the patient must accept the psychiatrist’s outpatient treatment plan. In a legal system where COT only arises following a hospital admission, a treatment plan refusal typically results in the maintenance of compulsory hospitalization. Although COT is less restrictive than hospitalization, patients on COT move into their community environment with significant limitations to their freedom. These limitations emerge because the patient must comply with a treatment plan, whether or not they agree with it [5]. As such, in line with the proportionality principle, COT should not be used solely because it constitutes a less restrictive means compared to hospitalization. Moreover, when facing patient disagreement regarding the outpatient intervention, the fact that the clinician considers a particular treatment plan to be good for the patient does not in itself constitute sufficient justification for the restriction of autonomy [10]. Indeed, discussion should follow outpatient treatment refusal to ascertain the need for further coercion. When applying COT, clinicians must be aware of the dangers of moving from soft paternalism to hard paternalism^1^ [33, 39].

When COT is deemed necessary and meets proportionality, psychiatrists should limit their COT treatment plan to evidence-based interventions and the actions that these entail. Additionally, the plan’s focus should only extend to the areas of life that are strictly necessary for achieving a defined therapeutic goal. Practitioners cannot ethically justify that COT status should indiscriminately prevent the patient from enjoying other rights such as privacy, intimacy, dignity, reputation, or personal identity. For example, it is reasonable that patients under COT may deny the community care team entry to their house based on the right to privacy, despite not being able to reject treatment. Indeed, privacy, reputation, and personal identity should be considered carefully in deliberations for instituting COT. COT entails a significant amount of monitoring and interference from health services for a person who most likely is already experiencing substantial scrutiny from family and friends [8]. Ultimately, an additional label of non-compliance or deviance may emerge, adding to the heavy burden of the stigma of mental illness[8]. These issues with COT may impact the rights of reputation and personal identity, areas where individuals with mental illness may already struggle. Any patient refusals based on these rights must be considered and addressed rather than readily interpreted as compliance defiance.

Another essential consideration concerning proportionality relates to time spent on COT. As the evidence for relapse prevention interventions has developed in recent years for conditions such as schizophrenia and bipolar disorder [40, 41], clinicians may feel inclined to use COT as a means to try to indefinitely prevent relapse in patients who have achieved remission but are thought to be at risk of disrupting their medication regime after discharge. However, the crucial question is whether such patients have the capacity to consent; if they do have capacity, coercion cannot be ethically justified, even despite good evidence for relapse prevention interventions (e.g., a manic patient who typically regains capacity after the acute episode and refuses relapse prevention interventions). Although some patients with an impaired decision-making capacity may need extended periods on COT (due to the specific characteristics of their mental illness and the time to effect of the intervention), extended treatment needs should not be assumed just because they have a diagnosis of a psychiatric disorder. Furthermore, mental illness does not necessarily indicate decision-making incapacity [33]. As the focus of COT is treatment, a reasonable expectation may be that the therapeutic intervention results in improving the decision-making capacity and the recovery of autonomy—particularly in the earlier phases of illness. Therefore, thorough, serial evaluations of decision-making capacity are paramount for minimising the time spent in a liberty-limiting situation.

In summary, clinicians should promote evidence-based therapeutic plans that are limited to essential measures in their scope. The goal of these plans involve causing the least possible impact in other areas of patients’ lives for the least amount of time. Therefore, there sould be a focus on the ethical imperative to minimize interference with patients’ enjoyment of other important rights.

#### The reciprocity principle

Upshur [16] outlined the reciprocity principle, stating that once public health action is necessary, an obligation arises to assist and compensate the individual when compliance with public health requests imposes certain burdens, such as a sacrifice of income or time [16].

Additionally, the decision to use COT imposes significant explicit and implicit limitations on patients’ autonomy within their own environment. Explicit limitations relate to the obligation to comply with the conditions of their treatment plan. Implicit limitations are more pervasive and include the sense of surveillance and the notion that failure to comply will result in compulsory hospitalization, often following a police referral [8].

The deliverance of treatment alone may be considered to fully satisfy the reciprocity principle. However, Engelhardt subsumed the principle of beneficence with the principle of autonomy [42]. This means that beneficence should not be based on an outside notion of best interests but should be accompanied by the individuals’ recognition of their own conception of good, as argued by Scholten and Gather [43]. This perspective may be challenging in psychiatry, as patients with mental illness may lack insight into their condition and, thus, may be unable to recognize it [33]. Consequently, COT should be balanced with a benefit that the patient can clearly perceive as such. As Upshur [16] argued, the implementation of coercive health measures accompanies sacrifices of income and time. Furthermore, submission to treatment is not without cost, namely in terms of medication, time availability, or even transportation. Therefore, if medication is necessary, it should be provided free of charge; moreover, if attending appointments is necessary, the transportation expenses should be compensated. An alternative to transportation compensation would include the use of widespread community teams reaching out to patients. These measures may appear utopian, but they do not seem implausible if considering other health programmes based on the same principles, such as the World Health Organization’s tuberculosis plan, which has experienced significant success [8].

#### The transparency principle

According to Upshur [16], the transparency principle entails ensuring that all participants are involved in the decision-making process and that the process is as clear as possible. According to this principle, the decision-makers are also obliged to provide information to justify their decision [15, 17]. Transparency plays an important role in the decision to implement COT. Firstly, transparency promotes accountability, which is essential for guaranteeing thoughtful deliberations, particularly when considering the curtailing of a fundamental right. Secondly, transparency allows decisions to be appealed; indeed, the appeal process is essential for reviewing previous decisions, thus allowing unjustified decisions to be revoked or, conversely, reinforcing the legitimacy of sound rulings. Accordingly, opacity promotes a further Kafkaesque atmosphere of obscurity, which may contribute to patients’ feelings of powerlessness and despair, especially because of the contact with the legal system.

Therefore, clinicians should seek to make their decisions as transparent and clear as possible, not only for the documentation but also for the patient. Such transparency means that a detailed analysis of the decision to initiate or maintain COT is required in every instance and that arguing abstractly that the patient meets the conditions ascribed by the law is insufficient. Instead, the analysis should detail how the patient’s mental state at a particular moment prevents them from exerting their autonomy and how it affects their decision-making capacity.

Evidence suggests that lack of insight into illness in schizophrenia has a neurobiological basis and, thus, represents more than simple defensive denial [20, 44–46]. Furthermore, Grisso and Appelbaum [47] demonstrated that in a hospital setting, one quarter to one half of the patients with schizophrenia lacked the capacity to make decisions based on four domains: evidencing a choice, understanding, reasoning, and appreciation. In community setting, less information is available to the clinician regarding the decision-making capacity of patients with schizophrenia. Nonetheless, assuming that—despite treatment and the improvement of psychotic symptoms—some patients will still be unable to recognize their illness seems plausible. Knowledge of these findings might incline clinicians to rely on preconceptions to assume decision-making incapacity and implement COT. Additionally, clinicians show other biases in terms of perceiving patients who agree with treatment as mentally competent and uncooperative patients as mentally incompetent [33]. As such, clinicians should always maintain some degree of scepticism about a patient’s decision-making inability. In line with this, according to Childress, clinicians should adopt a moral presumption of capacity and bear the burden of proving incapacity [33].

A direct and unquestioned association between the refusal of treatment or the existence of a mental illness diagnosis with incapacity to make decisions would restrict the autonomy of patients with mental illness who still maintain their decision-making ability. In turn, this situation would lead to an ethically indefensible position of patients with mental illness never being able to refuse treatment. Therefore, a thorough and transparent evaluation of decision-making capacity should be fundamental in the implementation of COT rather than a treatment refusal or the existence of a diagnosis of mental illness.

Despite the arguments regarding the relevance of decision-making assessments, it should be recognized that decision-making evaluations are demanding for the clinician, because the decision of ascribing a clinical verdict of decision-making incapacity may be complicated by the dimensional aspects of psychopathology. Symptom fluctuation, in terms of not only frequency but also intensity, may contribute to difficulties in translating the patient’s psychopathological state into a dichotomous decision regarding whether the patient has decision-making capacity or not. The decision must consider several informative inputs—varying from patient preferences and psychopathology to clinical history—that do not always form a clear-cut outcome, thus making this verdict extremely challenging clinically, ethically, and juridically. As such, the appreciation and documentation of the difficulties in assessment also constitute an important element of transparency in clinical decisions.

As another aspect of transparency, clinicians should document their attempted measures to persuade the patient to voluntarily adhere to the treatment plan. Indeed, this action would provide clarity on the decision-making process and strengthen the indispensability of the use of coercive measures to implement treatment.

It could be argued that considering this principle, as formulated above, constitutes a cynical stance because, ultimately, the patients’ views will be ignored if certain clinical criteria are met. Furthermore, not all participants have equal input into the decision-making process. However, we argue that the patient’s position should always be noted and documented. Acknowledging patients’ rights to formulate opinions and to hold views based on their values and beliefs is a sign of respect for their autonomy [26]. Therefore, patients’ assessment of the situation, as well as their reasoning, should contribute significantly to the final decision, even though it is realistic to recognize that the ultimate decision lies outside their scope of action.

## Conclusion

Upshur [16] outlined a set of principles to regulate ethical intervention in public health, which appear adaptable for ethically governing the implementation of compulsory treatment in the community. This framework not only provides a platform to enrich the theoretical debate but also facilitates ethical action within a medical and legal situation that has mixed evidence regardind its efficacy. Given the relevance of Upshur’s framework to the ethical application of COT, researchers and policymakers should investigate whether a more ethical solution to reduce the prevalence of compulsory regimes in psychiatry could be to improve public health policies. This solution of improving public health policies been suggested to address issues such as suicide or violence [34, 35, 48]. Indeed, coercion may be an immediate but ultimately ineffective solution for long-deferred problems and could be viewed as reflecting an inability to create conditions and skills for using less invasive treatment implementation options.

Addionally, adequate availability of well-resourced and innovative mental health services may provide an alternate solution to curtailing the individual rights of an already vulnerable population [49]. However, until these decisive policies are set in motion, ethical frameworks based on recognizable principles provide an useful assessment method to facilitate ethically sound decisions and actions regarding coercive treatment. The relevance of ethics in this context should not be underestimated, because ethical practices may contribute to reducing coercive measures and improve the quality of coercive treatment, thus offering a way to achieve a more dignified care within the current service and legal landscapes [50].

## Data Availability

Not applicable.

## Abbreviations

CDPD: Convention on the Rights of Persons with Disabilities
COT: Compulsory Outpatient Treatment
COVID-19: Coronavirus disease 2019
OCTET: Community treatment orders for patients with psychosis

## Footnotes

1 Soft and hard paternalisms represent two different paternalistic stances. In soft paternalism, an agent acts on behalf of another person with the goal of preventing substantially nonvoluntary conduct. Nevertheless, applying soft paternalistic actions may be morally complex because of the difficulty in deciding whether a person’s actions are substantially nonautonomous. In any case, it is morally accepted that we should protect people from harm caused to them by conditions beyond their control. Therefore, the sole purpose of soft paternalism is to prevent harmful consequences from an action that a patient did not choose with relevant autonomy. Indeed, soft paternalism does not pose a major conflict between the principles of respect for autonomy and beneficence. Conversely, hard paternalism involves actions that intend to prevent harm, or to benefit, a person, even if the person is aware of the risks and their choices are informed, voluntary, and autonomous. Therefore, hard paternalistic actions seize the individual’s autonomy by either restricting information or overruling the person’s informed and voluntary choices [39, p. 233].
